# Supplementary material for: An open-source framework for large-scale, flexible evaluation of biomedical text mining systems
Source: J Biomed Discov Collab. 2008 Jan 29;3:1. doi: 10.1186/1747-5333-3-1 (PMC2276192; doi:10.1186/1747-5333-3-1)
Supplement: Additional file 1 — Evaluation platform source code. The additional file contains the software evaluation framework discussed in this paper. The most current release can be downloaded from SourceForge.net [1]. See the accompanying README file for instructions on installation and use. Also included in the distribution is a collection of UIMA wrappers for some commonly used BioNLP tools and annotated corpora. [file 1747-5333-3-1-S1.zip › bionlp_uima_repository-1.0.1/docs/api/overview-frame.html]

Overview (Center for Computational Pharmacology Util/UIMA API)


All Classes

Packages
  
edu.uchsc.ccp.uima.ae.nlp.anaphora
  
edu.uchsc.ccp.uima.ae.nlp.ei.gene
  
edu.uchsc.ccp.uima.ae.nlp.ei.mutation
  
edu.uchsc.ccp.uima.ae.nlp.opendmap
  
edu.uchsc.ccp.uima.ae.nlp.parser
  
edu.uchsc.ccp.uima.ae.nlp.sentenceDetector
  
edu.uchsc.ccp.uima.ae.nlp.stemmer
  
edu.uchsc.ccp.uima.ae.nlp.tokenizer
  
edu.uchsc.ccp.uima.ae.util
  
edu.uchsc.ccp.uima.ae.util.converter
  
edu.uchsc.ccp.uima.ae.util.filter
  
edu.uchsc.ccp.uima.annotation
  
edu.uchsc.ccp.uima.annotation.syntactic
  
edu.uchsc.ccp.uima.cc.summary
  
edu.uchsc.ccp.uima.cc.summary.comparison
  
edu.uchsc.ccp.uima.cc.summary.opendmap
  
edu.uchsc.ccp.uima.mention
  
edu.uchsc.ccp.uima.util
  
edu.uchsc.ccp.uima.util.opendmap
  
edu.uchsc.ccp.util
  
edu.uchsc.ccp.util.external.abgene
  
edu.uchsc.ccp.util.external.abner
  
edu.uchsc.ccp.util.external.geniatagger
  
edu.uchsc.ccp.util.external.kex
  
edu.uchsc.ccp.util.external.lingpipe
  
edu.uchsc.ccp.util.external.opennlp
  
edu.uchsc.ccp.util.external.penn
  
edu.uchsc.ccp.util.external.stanfordparser
  
edu.uchsc.ccp.util.nlp
  
edu.uchsc.ccp.util.nlp.annotation
  
edu.uchsc.ccp.util.nlp.annotation.comparison
  
edu.uchsc.ccp.util.nlp.annotation.filter
  
edu.uchsc.ccp.util.nlp.annotation.printer
  
edu.uchsc.ccp.util.nlp.document
  
edu.uchsc.ccp.util.nlp.ei
  
edu.uchsc.ccp.util.nlp.ei.xml
  
edu.uchsc.ccp.util.nlp.mention
  
edu.uchsc.ccp.util.nlp.mention.comparison
  
edu.uchsc.ccp.util.nlp.parser.corpus
  
edu.uchsc.ccp.util.nlp.stats
  
edu.uchsc.ccp.util.nlp.stemmer
  
edu.uchsc.ccp.util.nlp.tokenizer
  
edu.uchsc.ccp.util.xml
